# XIAP-associating factor 1, a transcriptional target of BRD7, contributes to endothelial cell senescence

**DOI:** 10.18632/oncotarget.6962

**Published:** 2016-01-20

**Authors:** Jong-Ik Heo, Wonwoo Kim, Kyu Jin Choi, Sangwoo Bae, Jae-Hoon Jeong, Kwang Seok Kim

**Affiliations:** ^1^ Divisions of Radiation Effects, Korea Institute of Radiological and Medical Sciences, Seoul, Republic of Korea; ^2^ Research Center for Radiotherapy, Korea Institute of Radiological and Medical Sciences, Seoul, Republic of Korea

**Keywords:** XAF1, p53, ionizing radiation, DNA damage, cellular senescence, BRD7, Gerotarget

## Abstract

X-linked inhibitor of apoptosis (XIAP)-associated factor 1 (XAF1) is well known as an antagonist of XIAP-mediated caspase inhibition. Although XAF1 serves as a tumor-suppressor gene, the role of XAF1 in cellular senescence remains unclear. We found that XAF1 expression was increased by genotoxic agents, such as doxorubicin and ionizing radiation in pulmonary microvascular endothelial cells, consequently leading to premature senescence. Conversely, downregulation of XAF1 in premature senescent cells partially overcame endothelial cell senescence. p53 knockdown, but not p16 knockdown, abolished senescence phenotypes caused by XAF1 induction. XAF1 expression was transcriptionally regulated by Bromodomain 7 (BRD7). XAF1 induction with interferon-gamma (IFN-γ) treatment was abrogated by BRD7 knockdown, which resulted in blocking interferon-induced senescence. In lung cancer cells, XAF1 tumor suppressor activity was decreased by BRD7 knockdown, and inhibition of tumor growth by IFN-γ did not appear in BRD7-depleted xenograft tumors. These data suggest that XAF1 is involved in BRD7-associated senescence and plays an important role in the regulation of endothelial senescence through a p53-dependent pathway. Furthermore, regulation of the BRD7/XAF1 system might contribute to tissue or organismal aging and protection against cellular transformation.

## INTRODUCTION

Normal somatic cells cultured *in vitro* have a limited ability to divide before entering a state of irreversible proliferative arrest termed replicative senescence [1]. Replicative senescence is induced by telomere attrition, response to activated oncogenes and DNA damage, deregulated nutrient sensing, loss of proteostasis and epigenetic alterations during aging [2-4].

Irreversible growth arrest is induced in primary cells through the expression of activated oncogenes such as Ras [5] or by activation of tumor suppressor genes [6]. Numerous studies have implicated the tumor suppressors p53, p16 and Rb as common major effectors of cellular senescence in normal somatic cells [7-8]. In this study, we used genetic approaches to search for previously unexplored senescence regulators in endothelial cells, particularly those involved in BRD7-associated senescence.

Bromodomain 7 (BRD7) is a unique component of the SWI/SNF polybromo-associated BRG1-associated factor (PBAF) complex that contributes to proliferation regulation [9]. It was originally identified as a gene whose mRNA was downregulated in nasopharyngeal carcinoma [10]. Recent studies have implicated BRD7 as a regulator of replicative senescence based on the induction of resistance to Ras-induced senescence by BRD7 depletion [11] or the induction of oncogene-induced senescence through BRD7 interaction with p53 and p300 [12].

Bromodomains are evolutionally conserved domains that have specific binding affinity for acetylated lysines on histone N-terminal tails [13]. Although the function of bromodomains still requires further investigation, bromodomain proteins modulate chromatin remodeling and modification, thereby facilitating accession of transcription factors to chromatin [14-16].

X-linked inhibitor of apoptosis (XIAP)-associated factor 1 (XAF1) directly and indirectly regulates p53-mediated apoptosis as a tumor suppressor gene. XAF1 is expressed ubiquitously in all healthy adult and fetal tissues, but is lost or reduced in a variety of cancer cell lines because of the aberrant promoter hypermethylation of its gene [17-18]. XAF1 was originally identified as a nuclear protein that has the ability to bind XIAP and antagonize the ability of XIAP to suppress caspase activity and cell death [18]. XAF1 can also induce apoptosis through an alternative pathway by enhancing TNF-alpha independently of interaction with XIAP [19].

Despite previous reports showing the implications of XAF1 in p53-mediated apoptosis in cancer, the molecular and cellular effects of XAF1 in primary normal vascular endothelial cells have not been examined. In the current study of the transcriptional regulation by BRD7 in endothelial cell senescence during irradiation, we have found a correlation between XAF1 and BRD7 in radiation-induced senescence. In this study, we demonstrate that XAF1 plays a crucial role in cellular senescence through transcriptional regulation by BRD7 in human endothelial cells.

## RESULTS

### XAF1 expression increases during DNA damage-induced senescence in endothelial cells

**Figure 1:**
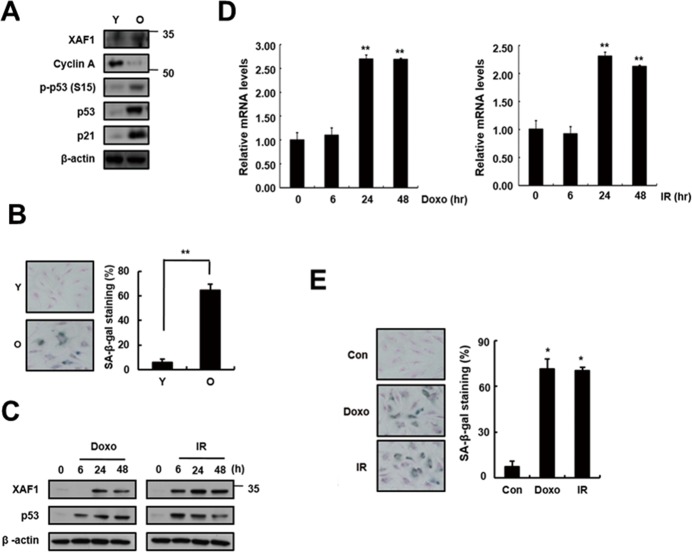
Increase in XAF1 expression in cellular senescence **A.** XAF1 protein expression levels XAF1 were analyzed by Western blotting with antibodies against XAF1 and GAPDH (loading control) in aged HMVECs. The levels of cyclin A, phospho-p53(S15), p53, p21 and β-actin proteins were detected by Western blot analysis. The figure shows representative data from three independent experiments. **B.** Percentages of SA-β-gal positive cells analyzed in young and old HMVECs. **p < 0.01 versus the young cells. **C.** XAF1 and p53 protein expression levels were detected by Western blot analysis. **D.** HMVECs treated with doxorubicin (Doxo, 1 μM) or ionizing radiation (IR, 4 Gy). The cells were incubated for 0, 24 or 48 h, and the XAF1 mRNA expression levels were measured by semi-quantitative PCR and real-time PCR analyses. Values are expressed as the mean ± SD of three independent experiments. **p < 0.01 versus the control group without Doxo or IR treatment. **E.** After treatment with Doxo or IR for 4 days, Percentages of SA-β-gal positive cells analyzed in young and premature senescent cells. *p < 0.05 versus the control cells. Y, young cells; O, old cells.

To investigate whether XAF1 is associated with cellular senescence in pulmonary endothelial cells, we examined XAF1 expression levels in young and old cells by semi-quantitative PCR (q-PCR), real-time PCR and Western blot analysis. Senescent cells are known to be resistant to mitogen-induced proliferation, express SA-β-gal and have a characteristically enlarged and flattened morphology. Using serial passaging with trypsinization, senescent cells (also referred to herein as ‘old cells’) were obtained and characterized by p53/p21 activation and SA-β-gal staining (Figure 1A, 1B). XAF1 protein levels were upregulated 3-fold or more in the old endothelial cells (Figure 1A). XAF1 expression was increased by DNA damaging agents, such as doxorubicin (Doxo) and ionizing radiation (IR) [20-21], which induced cellular senescence in endothelial cells (Figure 1C). These cells showed senescent phenotypes that distinguished them from the early-passage cells. Doxo or IR treatment increased XAF1 protein and mRNA levels in a time-dependent manner (Figure 1C, 1D) and resulted in morphological changes in old cells and increases in SA-β-gal staining activity (Figure 1E). These results imply that XAF1 upregulation is implicated in cellular senescence in old and IR-treated endothelial cells.

### Effects of XAF1 upregulation on cellular senescence in HMVECs

**Figure 2:**
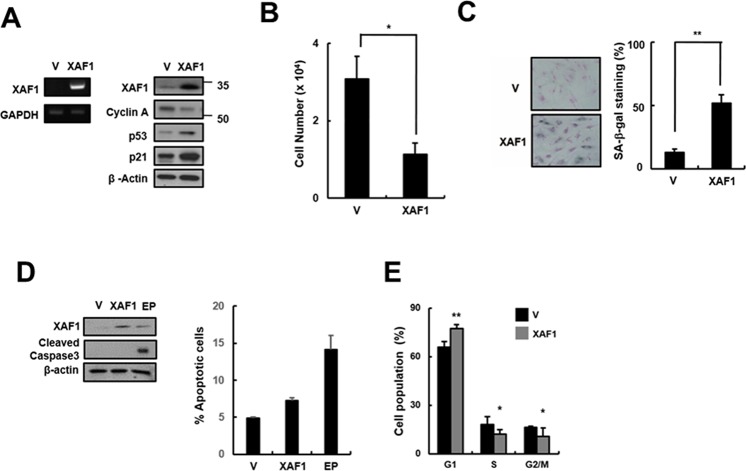
Effects of XAF1 on the upregulation of cellular senescence in young HMVECs **A.** Young cells were transduced with XAF1 or negative control lentiviruses and incubated for 3 days. XAF1 mRNA expression levels were measured by semi-quantitative PCR and XAF1, cyclin A, p53 and p21 protein levels were detected by Western blot analysis. **B.** Cell proliferation was measured by cell counting and **C.** the percentages of SA-β-gal positive cells were analyzed. *p < 0.05 and ** < 0.01 *versus* the vector group. **D.** XAF1-transduced cells were stained with Annexin V-FITC and propidium iodide (PI). To measure apoptosis, fluorescence intensities of Annexin V-FITC were analyzed by flow cytometry. Etoposide (EP, 100 μM) was used as a positive treatment. Cleaved caspase 3 activity was analyzed by Western blot. **E.** Cell cycle profiles were analyzed by PI staining and flow cytometry. Values are expressed as the mean ± SD of three independent experiments. Representative data from three independent experiments are shown. *p < 0.05 and **p < 0.01 *versus* the vector group. V, control vector lentivirus-transduced cells; XAF1, XAF1 lentivirus-transduced cells.

Because XAF1 expression levels were increased in senescent cells and by treatment with the DNA damage-related senescence inducers Doxo and IR, we tested whether XAF1 overexpression has an impact on cellular senescence in HMVECs. HMVECs were transduced with XAF1 lentivirus, and senescence markers in XAF1-overexpressing cells were examined. XAF1 upregulation caused a decrease in cell proliferation and an increase in SA-β-gal staining compared with the control lentivirus-transduced cells (Figure 2B, 2C). Increased XAF1 expression induced the expression of cell cycle regulators, such as p53 and p21, and inhibited the expression of Cyclin A (Figure 2A), resulting in G0/G1 cell-cycle arrest but not apoptosis (Figure 2D, 2E). Taken together, these results suggest that XAF1 might play an important role in inducing senescence in HMVECs.

### XAF1 knockdown reverses premature senescence in HMVECs

**Figure 3:**
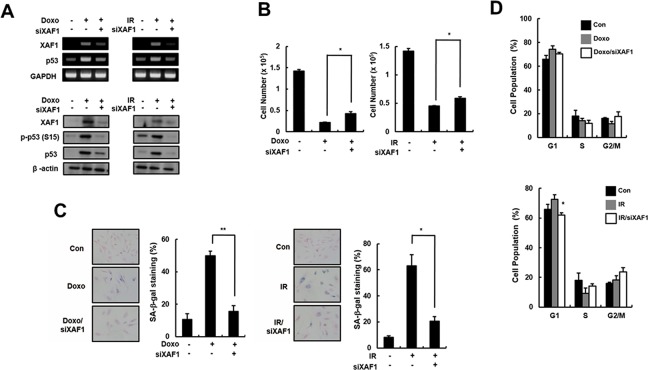
Partial reverse of premature senescence by XAF1 downregulation in HMVECs **A.** HMVECs treated with doxorubicin (Doxo, 1 μM) or ionizing radiation (IR, 4 Gy). The cells were incubated for 48 h before transfection with XAF1 siRNAs (siXAF1) or negative control siRNAs. XAF1 knockdown in Doxo- or IR-treated cells was confirmed by RT-PCR (upper panel) and Western blot analysis (lower panel). **B.** Effects of XAF1 knockdown on premature senescence by Doxo or IR in XAF1 siRNAs-transfected cells were examined by cell proliferation and **C.** SA-β-gal staining (100x). **D.** Cell cycle profiles were analyzed by PI staining and flow cytometry. The figure shows representative data from three independent experiments. Values are expressed as the mean ± SD of three independent experiments. *p < 0.05 and **p < 0.01 *versus* the siRNA control group.

To investigate the role of XAF1 in cellular senescence, HMVECs were treated with two DNA damage agents (Doxo and IR) that trigger premature senescence. XAF1 mRNA and protein levels were downregulated by gene silencing using XAF1 siRNAs in the senescent cells. The Doxo- and IR-induced increase in p53 expression and activation was reduced in the XAF1 knockdown cells (Figure 3A). Decreased cell proliferation in Doxo- or IR-treated cells was partially recovered by XAF1 downregulation (Figure 3B). Repression of XAF1 levels in senescent cells caused morphological changes similar to young cells and a decrease in SA-β-gal activity (Figure 3C). Additionally, XAF1 downregulation in premature senescent cells decreased the population of cells in the G1 phase and increased the population of cells in the S and G2/M phases (Figure 3D). These results suggest that XAF1 knockdown in old cells partially reverses senescence phenotypes.

### XAF1 induces cellular senescence through a p53 signaling pathway

**Figure 4:**
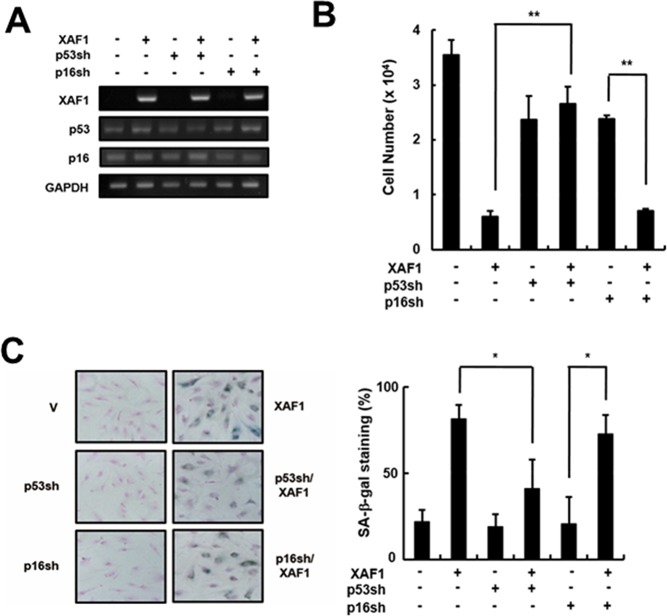
Effects of p53 or p16 knockdown on cell growth arrest induced by XAF1 **A.** Cells were transduced with p53- or p16-shRNA retroviruses and incubated for 2 days. P53 and p16 knockdown was confirmed by RT-PCR analysis. The p53- or p16-downregulated cells were transduced with XAF1 or negative control lentiviruses and incubated for 2 days. Cell proliferation was measured by **B.** cell counting for 2 days and **C.** SA-β-gal staining (100x). Values are expressed as the mean ± SD of three independent experiments. Representative data from three independent experiments are shown. *p < 0.05 and **p < 0.01 *versus* XAF1 overexpression. p53sh, p53-shRNA retrovirus transduced cells; p16sh, p16-shRNA retrovirus transduced cells.

The p53 and Rb tumor suppressor pathways play critical roles in activating senescence response [22]. It is reasonable to expect that XAF1 activates specific signaling pathways to engage p21 via p53 protein and/or p16/Rb proteins. To determine which pathway is involved in XAF1-mediated cellular senescence, we knocked down p16 and p53 using shRNA retroviruses in HMVECs and measured the effects of XAF1 on cellular senescence (Figure 4A). The p16 knockdown cells showed a decrease in cell proliferation by overexpressing XAF1 similar to the control cells. In contrast, XAF1 overexpression had no effect on cell proliferation in the p53 knockdown cells (Figure 4B). By measuring the SA-β-gal activity, we demonstrated that p53 knockdown inhibited XAF1-induced cellular senescence but p16 knockdown did not (Figure 4C). Therefore, these results suggest that XAF-induced cellular senescence might be mediated through a p53-dependent pathway.

### The role of BRD7 in XAF1 transcriptional regulation

**Figure 5:**
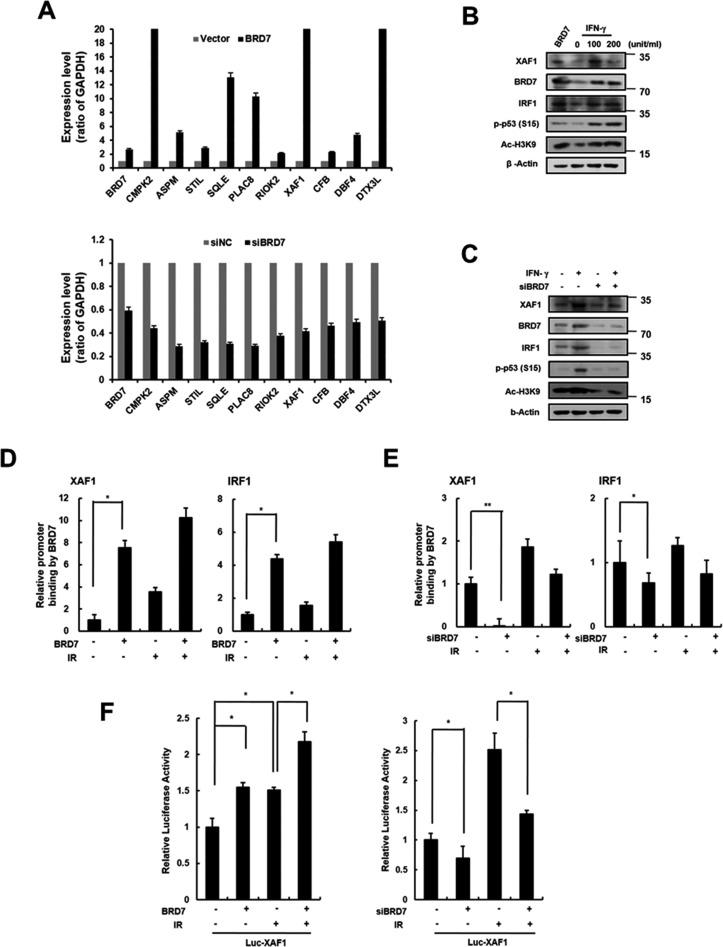
Transcriptional regulation of XAF1 by BRD7 **A.** XAF1 was upregulated with BRD7 lentivirus or downregulated with BRD7 siRNAs in HMVECs. BRD7, CMPK2, ASPM, STIL, SQLE, PLAC8, RIOK2, XAF1, CFB, CBF4 and DTX3L expression levels were analyzed by real time-PCR. Each of the expression levels were normalized to GAPDH expression levels. **B.** Cells were transduced with BRD7 lentivirus or treated with 0, 20 or 100 ng/ml interferon-gamma (IFN-γ) for 24 h. XAF1, BRD7, IRF1, phospho-p53 and Ac-H3K9 protein levels were detected by Western blot analysis. **C.** Cells were transfected with BRD7 siRNAs and treated with IFN-γ for 24 h. XAF1, BRD7, IRF1 and phsopho-p53 protein levels were detected by Western blot analysis. **D.** Cells were transduced with BRD7 lentivirus or transfected with BRD7 siRNAs. Each of cells was treated with 4 Gy IR for 24 h. Chromatin immunoprecipitation (ChIP) assay was performed using the anti-BRD7 antibody and the immunoprecipitated DNA was amplified using primers for p53, IRF1 or XAF1. **E.** The cells were transfected with the luciferase reporter constructs containing the XAF1 promoter. XAF1 promoter activity in BRD7-expressed cells was determined by luciferase analysis. *p < 0.05 and **p < 0.01 *versus* the siRNA control group.

BRD7 acts a transcriptional cofactor of promoter activity for a subset of p53 target genes [23]. We recently identified BRD7 as a potential regulator of DNA damage-induced senescence in endothelial cells. Using DNA microarray analysis, we compared the gene expression profiles from BRD7 down- or upregulated cells to identify DNA damage-related genes transcriptionally regulated by BRD7. As shown in Figure 5A, we selected several genes that were potentially regulated by BRD7 and confirmed the expression of each gene in BRD7 down- or upregulated HMVECs using real time PCR. One of these genes, XAF1, was verified to be a BRD7-regulated gene. Interferon regulatory factor 1 (IRF1) is known to be an XAF1 transcription factor and is induced by interferon stimulation. Similar to cells treated with interferon-gamma (IFN-γ), BRD7 could induce XAF1 expression (Figure 5B). To test whether BRD7 exerts a direct influence on XAF1 expression, we measured XAF1 expression when BRD7-downregulated cells were treated with IFN-γ. Despite the presence of IFN-γ stimulation, XAF1 was downregulated by BRD7 depletion, suggesting that BRD7 may mediate a direct effect on XAF1 expression (Figure 5C). Using chromatin immunoprecipitation (ChIP) (Figure 5D, 5E) and luciferase assays (Figure 5F), we further confirmed transcriptional regulation of XAF1 by the BRD7 protein. Consequently, the BRD7 protein increased the promoter activity of the XAF1 gene. Therefore, BRD7 is required for XAF1 transcriptional expression.

### XAF1 as a tumor suppressor gene in lung cancer cells

**Figure 6:**
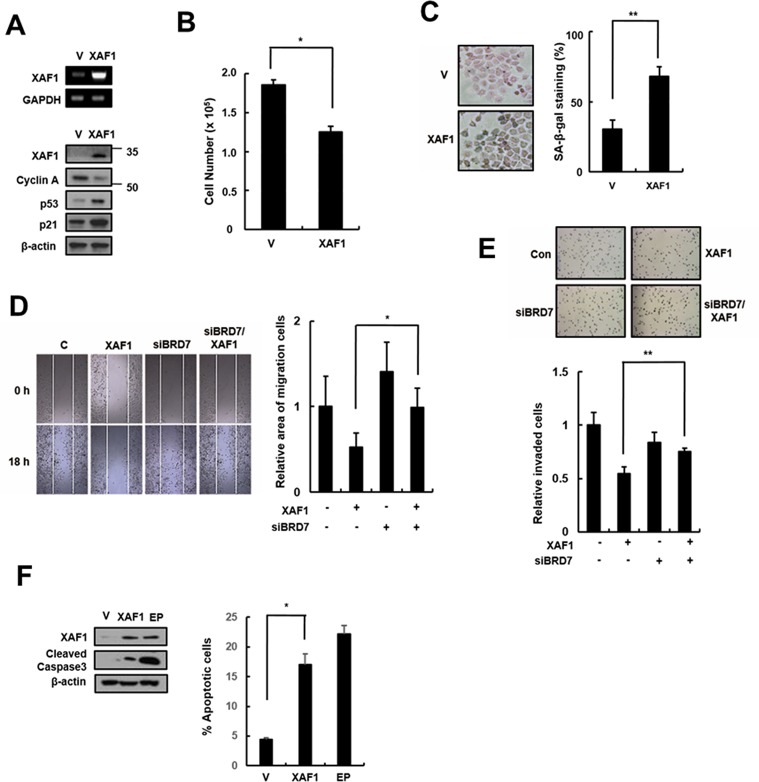
Effects of XAF1 expression in lung cancer cells **A.** XAF1 was upregulated in A549 lung cancer cells and its expression levels were confirmed by semi-quantitative RT-PCR and western blot analysis. Cell proliferation was measured by **B.** cell counting for 2 days and **C.** SA-β-gal staining (100x) and the percentage of SA-β-gal positive cells were analyzed in XAF1 upregulated cells **C.**. *p < 0.05 and **p < 0.01 *versus* the vector group. **D.** Cell migration and **E.** invasion assays were performed with the XAF1 upregulated cells. *p < 0.05 and **p < 0.01 *versus* XAF1 overexpression. **F.** Cleaved caspase 3 activity was analyzed by Western blot. Percentage of apoptosis cells was analyzed by flow cytometry with Annexin V-FITC. Etoposide (EP) was used as a positive treatment. *p < 0.05 *versus* vector group.

XAF1 is a tumor suppressor that is frequently silenced in many human cancers, but it can inhibit cell proliferation and induce apoptosis in some cancer types. XAF1 increases sensitivity to IR in lung cancer cells, but its effect in lung cancer remains unclear. We tested the cellular effects of XAF1 expression in the human lung cancer cell line A549. Similar to its effect in HMVECs, XAF1 expression increased p21 expression (Figure 6A), induced cell cycle arrest (Figure 6B) and increased SA-β-gal staining (Figure 6C) in A549 cells. To investigate whether BRD7 acts as critical regulator of XAF1 in lung cancer cells, we examined migration and invasion by transfecting BRD7 siRNAs into cells overexpressing XAF1. XAF1 decreased migration and invasion ability in lung cancer cells, although the increase in XAF1 expression, migration and invasion was not reduced by BRD7 depletion (Figure 6D, 6E). Unlike in HMVECs, XAF1 induced apoptosis in lung cancer cells (Figure 6F). Therefore, these results suggest that BRD7 expression plays a critical role in XAF1 tumor suppression.

### Effects of BRD7-regulated XAF1 in animal model tumors

**Figure 7:**
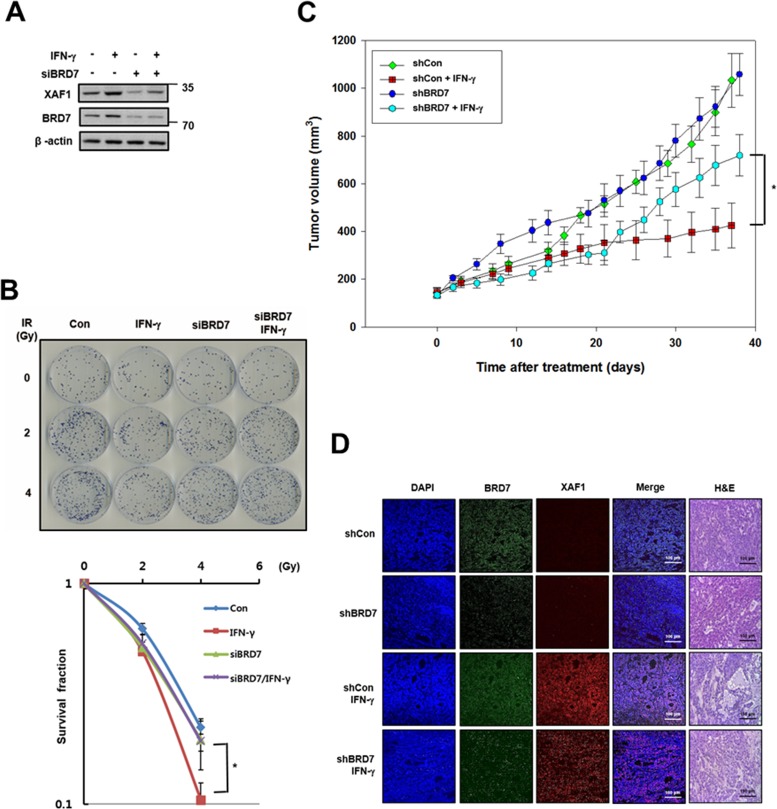
Effects of BRD7-dependent XAF1 expression *in vivo* **A.** A549 cells were transfected by BRD7 siRNA (siBRD7) or scramble siRNA and then each of the cells was treated with IFN-γ (100ng/ml). Expression of XAF1 and BRD7 proteins were confirmed by Western blotting. **B.** Each of the cells was irradiated with 0, 1, 2 and 4 Gy of IR, and incubated about 2 weeks for colony formation. Cell survival values were normalized to those of the unirradiated cells. *p < 0.05 *versus* BRD7-depleted group. **C.** Effect of IFN-γ administration on growth of A549 tumor xenografts in nude mice. Nude mice (*n* = 10 per group) were implanted subcutaneously with A549 and groups of mice received vehicle or IFN-γ (1 × 10^5^ unit) three times for a week. Tumor size was assessed by direct caliper measurement. Data are means ± SE, *n* = 10. Tumor volume between shCon and shBRD7 in IFN-γ treated group was statistically significantly different at *p < 0.05 by two-sided Student's *t*-test. **D.** Xenograft tumors were embedded with paraffin, were serially sectioned and stained with hematoxylin and eosin (H&E). The tissue samples were immunostained with XAF1 and BRD7 antibodies and observed with fluorescence microscope.

As shown in Figure 5, XAF1 is regulated by IFN-γ, meaning that IFN-γ is a good tool for inducing XAF1. Because IFN-γ-induced XAF1 is a potential radio-sensitizer in lung cancer cells [24], we tested whether BRD7 can affect the radio-sensitizing activity of IFN-γ-induced XAF1. After IFN-γ treatment, XAF1 protein levels were regulated by BRD7 depletion (Figure 7A). Following IR, a colony forming assay showed that IFN-γ treatment decreased clonogenic cell survival in A549 lung cancer cells but that BRD7 depletion inhibited the decrease in clonogenic cell survival induced by IFN-γ treatment (Figure 7B). With INF-γ treatment, we confirmed that BRD7 regulates the tumor suppression activity of XAF1 *in vivo*. A549 cells were transduced with BRD7 or control shRNA lentiviruses, selected with puromycin and then established as A549 xenografts in nude mice. The change in tumor volume was monitored three times a week after INF-γ treatment. Tumor growth was significantly suppressed by INF-γ treatment, but BRD7 depletion prevented the decrease in tumor growth by INF-γ treatment (Figure 7C). Xenograft tumors were processed for histology and stained with hematoxylin and eosin (H&E) or immunostained with XAF1 and BRD7 antibodies (Figure 7D). H&E staining demonstrated that tumor necrosis induced by IFN-γ treatment was decreased in the BRD7-depleted group and that IFN-γ-induced XAF1 expression was not shown in BRD7-depleted tumors. These observations indicate that INF-γ-induced XAF1 and that XAF1 tumor suppression is determined by BRD7 *in vitro* as well as *in vivo*.

## DISCUSSION

Along with surgery and chemotherapy, radiation therapy (radiotherapy) is one of the most important methods for cancer treatment. Currently, more than half of all cancer patients receive radiation therapy. Radiation kills cancer cells by damaging their DNA, but it can also damage normal cells and some tissue types. It is not easy to balance the two conflicting effects during cancer therapy. Exposure to ionizing radiation leads to adverse physiological responses, such as cancer, gastrointestinal failure, bone marrow failure and accelerated aging, but these risks are not exclusively a result of radiotherapy. Generally, the radiation-associated excess relative risks are lower than excess absolute risks in medically exposed individuals [25]. Therefore, it is important to improve radiotherapy to increase the curative and survival rates for patients.

Thus, we have to consider endothelial cell function in the opposite point of view in cancer and vascular diseases. In cancer biology, endothelial cells are homeostatic factors for regulating angiogenesis-dependent tumor growth [26]. Microvascular damage regulates tumor cell response to radiation, and radiation-induced vascular damages enhance rapid apoptosis in tumors and prevent the recurrence of tumors after radiation treatment [27]. In this study, increased XAF1 was associated with the induction of a variety of senescent phenotypes, including growth arrest by p53 activation and an increase in senescence-associated β-galactosidase (SA-β-gal) staining in both pulmonary endothelial cells and lung cancer cells (Figures 2, 7). Therefore, XAF1 may be a potential inhibitor of angiogenesis and tumor growth because XAF1 binds directly to p53 and induces p53-mediated apoptosis in cancer.

In contrast, senescent cells have been suggested to contribute to aging-associated pathologies via the accumulation of non-dividing cells and alterations in tissue structure and function [8]. Therefore, endothelial cell senescence contributes to the age-related decline of vessel structure and the progression of various age-associated vascular diseases. XAF1 induces endothelial cell senescence, suggesting that XAF1 may contribute to vascular aging and the development of aging-associated vascular diseases.

Recently, several groups have reported that radiation induces senescence in endothelial cells. Endothelial cell senescence is associated with vascular damage and vascular disease *in vivo*. DNA damaging agents, such as ionizing radiation, can accelerate the onset of endothelial cell senescence through numerous changes in protein expression [28]. We have examined the biological networks that are affected by ionizing radiation from epigenetic alteration. Aging is associated with profound epigenetic changes, resulting in extensive remodeling of gene-expression profiles and disturbances in broad epigenomic landscapes. The relationship between epigenetic changes and aging has been confirmed by changes in DNA methylation levels [29], histone levels and their post-translational modifications [30] and transcriptional gene silencing by non-coding RNAs [31]. Among them, histone modifications directly contribute to nuclear compartmentalization for the formation of chromatin domains, such as heterochromatin and euchromatin, which are transcriptionally silent and active, respectively [32]. Specifically, with respect to histone modifications and aging, increased H3K9 acetylation leads to shortened life span and premature aging-like phenotypes in response to DNA damage [33]. Conversely, H3K9 di- and tri-methylation are reduced by DNA damage and replicative aging [34]. Furthermore, H3K4 demethylation can induce the transcriptional silencing of the retinoblastoma target genes in senescent cells [35] and methylated H3K36 can regulate cell proliferation and senescence via the p53 and Rb pathways [36].

BRD7 is a selected marker for both cellular senescence and DNA damage response for epigenetic studies with radiation-induced senescence. BRD7 participates in chromatin remodeling by binding to acetylated histones and regulates the expression of genes as a transcription factor. Using genechip arrays and chromatin immunoprecipitation analysis, we have newly founded that XAF1 is transcriptionally increased by BRD7 during radiation exposure (Figure 4).

Although there is not known for epigenetic alteration by BRD7, the reversibility of epigenetic modifications and gene expression by BRD7 inhibition will provide a new possibility for therapeutic interventions in age-related diseases as well as radiation damage regulation.

## MATERIALS AND METHODS

### Materials

Human lung microvascular endothelial cells (HMVECs) and endothelial cell basal medium-2 (EBM-2) supplemented with endothelial growth medium 2 (EGM-2) were obtained from Lonza (Walkersville, MD, USA). Roswell Park Memorial Institute medium (RPMI 1640), fetal bovine serum (FBS), 100 U/ml of penicillin and 100 μg/ml of streptomycin were purchased from Welgene (Daegu, Korea). Antibodies against XAF1 (sc-19194), BRD7 (sc-376179), IRF-1 (sc-497), cyclin A (sc-239), p53 (sc-126), p21 (sc-397) and beta-actin (sc-81178) were from Santa Cruz Biotechnology Inc. (Santa Cruz, CA, USA), and antibodies against phospho-p53 (Ser15) (#9248), acetylated lysine 9 of histone H3 (AcH3K9) (#9649) and cleaved Caspase3 (#9661) were from Cell Signaling Technology Inc. (Danver, MA, USA). Horseradish peroxidase-conjugated secondary rabbit, mouse and goat antibodies were from Bethyl Laboratories, Inc. (Montgomery, TX, USA). XAF1 siRNA (AUGAAGGAAGAAUUUCUGGAUUUCC) and Lipofectamine 2000 reagent were from Invitrogen Life Technologies Inc. (Carlsbad, CA, USA), and the pLKO.1-shBRD7 vectors (TRCN0000360087) were from Sigma-Aldrich (St. Louis, MO, USA). BRD7 siRNA (L-020297-00) was from GE Dharmacon (Lafayette, CO, USA). The PCR primers for XAF1, p53, p16 and GAPDH were obtained from Bioneer (Daejeon, Korea). We obtained bromo-chloro-indolyl-galacto-pyranoside (BCIG; X-gal) from Duchefa (Haarlem, Netherlands), a total RNA isolation (TRI) solution from Molecular Research Center, Inc. (Montgomery, CA, USA) and a reverse transcription-polymerase chain reaction kit from the Promega Corp. (Madison, WI, USA).

### Cell culture

HMVECs in EBM-2 supplemented with EGM-2 and A549s in RPMI 1640 were plated at a density of 2 × 10^5^ cells per 100 mm culture plate and cultured at 37°C in a 5% CO_2_ humidified incubator. When the subcultures reached 80% confluence, serial passaging was performed by trypsinization. For experiments, cells were used in either passage 5-7 (PD < 24; young) or passage 12-15 (PD > 48; old).

### Induction of cellular senescence by adriamycin treatment and γ-ray irradiation

HMVECs and A549s (2 × 10^5^ cells) were seeded in 60-mm culture dishes and incubated for 24 h in culture media. The cells were washed with media and treated with 1 μM doxolubicin (Doxo) for 4 h. After discarding the media containing the Doxo, the cells were washed 3 times with media and incubated in culture media for the indicated times. For ionizing radiation (IR) treatment, cells were exposed to γ-rays with a 137 Cs γ-ray source (Atomic Energy of Canada, Ltd.) at a dosage of 3.81 Gy/min.

### Virus preparation and transduction

For XAF1 overexpression, the XAF1 transcript was cloned into a pLenti6/V5-TOPO vector (Life Technologies Corporation, Carlsbad, CA, USA). Cells were transduced with XAF1 lentivirus for 48 h. p16 and p53 shRNA retroviruses were prepared by transfection of the pRetroSuper-p53sh and pRetroSuper-p16sh vectors. After incubation for 3 days, media were collected and centrifuged at 1,650 *g* for 10 min. The viral solution was filtered with 0.45-μm filter membranes and concentrated with a Vivaspin 20 (Sartorius, Goettingen, Germany).

### Cell counting

Cells were harvested by trypsin-EDTA treatment and stained with 0.1% trypan blue, and the cell number was determined using a hemocytometer.

### Clonogenic cell survival assay

A clonogenic cell survival assay was performed by following a standard protocol [37].

### Invasion and migration assay

Cell invasive activities were assessed using a 12-well Transwell^®^ insert from Corning Life Sciences (Lowell, MA, USA). Cells were seeded in the absence of serum at a concentration of 2 × 10^5^ cells in the upper chamber. The lower chamber was filled with culture medium supplemented with 10% FBS. After 24 hours, invaded cells that had passed through the Matrigel were fixed with methanol, stained with 0.1% crystal violet, and counted under a light microscope (200× magnification). For the migration assay, cells were seeded at a density of 1 × 10^6^ cells in a 12-well dish. Scratch wounds were created with pipette tips through the confluent cells. Migration/wound-healing was observed after 24 h.

### Immunofluorescence staining

Cells were fixed with 3.7% para-formaldehyde in PBS for 3 min and permeabilized in PBS containing 0.5% Triton X-100 for 5 min. The cells were incubated with an antibody specific to XAF1 (1:200) for 2 h and then with Alexa Fluor^®^ 594 donkey anti-Goat IgG (1:300) (Life Technologies, Carlsbad, CA, USA) for 30 min. The nuclei were stained with 0.1 μg/ml of 4′,6-diamidino-2-phenylindole (DAPI) for 5 min. Images were obtained using a fluorescence microscope.

### Senescence-associated β-galactosidase (SA-β-gal) staining

Cells were plated at a density of 4 × 10^4^ cells in 35 mm culture dishes, washed with PBS and fixed with 3.7% (v/v) paraformaldehyde in PBS for 3 min at room temperature. The presence of SA-β-gal activity was determined by incubating the cells with a solution containing 40 mM citric acid-sodium phosphate (pH 6.0), 150 mM NaCl, 2 mM MgCl_2_, 5 mM potassium ferricyanide, 5 mM potassium ferrocyanide and 1 mg/ml X-gal for 17 h at 37°C. The percentage of blue cells per 100 cells observed under a light microscope was measured.

### Total RNA extraction and reverse transcription-polymerase chain reaction

RNAs were prepared from cells using the TRI solution according to the manufacturer's instructions. cDNA was prepared from RNA (2 μg) using 2.5 μM oligo-dT primers, 1 mM dNTPs and moloney murine leukemia virus (MMLV) reverse transcriptase. XAF1, p53 and p16 were amplified from the cDNA with AccuPower^®^ PCR PreMix (Bioneer Inc., Daejeon, Korea) and gene-specific oligonucleotides. GAPDH primers were used to estimate the amount of RNA in each sample. PCR products were separated on 1.2% agarose gels, and the DNAs were visualized by SYBR Green staining.

### Protein extraction and western blot analysis

Cells were washed with PBS and lysed in 70 μl of RIPA buffer (25 mM Tris-HCl, pH 7.6, 150 mM NaCl, 1% Triton X-100, 0.5% sodium deoxycholate, 0.1% SDS, 1 mM sodium vanadate, 5 mM NaF and protease inhibitor). The proteins were quantified with the bicinchoninic acid (BCA) method using bovine serum albumin as a standard. Proteins (30 μg) were separated by 10% SDS-polyacrylamide gel electrophoresis and then transferred to nitrocellulose membranes. The membranes were incubated overnight at 4°C with specific antibodies. After washing 5 times in Tris-buffered saline containing 1% Tween 20 (TTBS), the membranes were incubated with horseradish peroxidase-conjugated secondary antibodies. The proteins were visualized using enhanced chemiluminescence (GE Healthcare Life Sciences, Buckinghamshire, United Kingdom).

### Chromatin immunoprecipitation

Cells (1 × 10^6^) were plated on 100 mm-culture plates and incubated under various conditions. Cells were treated with 1% formaldehyde for 10 min at room temperature to crosslink protein–DNA complexes. Cells were harvested, washed three times in 1× PBS and lysed in SDS with 50 mM Tris-HCI (pH 8.1) and 1 mM EDTA. Cells were sonicated for 1 hour, and the supernatants were evenly split for incubation with the primary antibodies. Reactions with a final volume of 1 ml in dilution buffer with 2 μg of the appropriate primary antibody were rotated overnight at 4°C. Then, 60 μl of Protein A beads was added to each reaction and rotated for 1 h at 4°C. The beads were washed three times. The eluted supernatants as well as input DNA samples were then uncrosslinked via incubation at 65°C for 4 h. The DNA was then ethanol precipitated and treated with Proteinase-K for 30 min at 37°C. The samples were then extracted with phenol:chloroform, precipitated in ethanol and resuspended in 20 μl of distilled water. Promoter binding activity was measured by real time-PCR analysis with specific promoter primers. The promoter primer sequences are as follows: (1) XAF1 promoter, (forward primer, 5′-cctgagtagctgggattata-3′ and reverse primer, 5′-ctgggcagtctgtcaatatc-3′) and (2) IRF1 promoter (forward primer, 5′-aatcccgctaagtgtttgga-3′ and reverse primer, 5′-agtggaagagggaagaaggc-3′).

### Flow cytometry

Cells were harvested, washed twice with PBS and fixed with 70% ethanol at −20°C for 1 h. After washing the cells with PBS containing 2% FBS and 0.01% CaCl_2_, RNase (0.5 μg/ml) was added and incubated with the cells at 37°C for 30 min. Then, propidium iodide (50 μg/ml) was added and incubated at room temperature for 20 min. The intracellular propidium iodide fluorescence intensity of each population of 10,000 cells was measured using a Becton-Dickinson FACSCalibur flow cytometer.

### *In vivo* xenograft assay

Nude mice (Balb/c, 5-week-old males) were obtained from Orientbio, Inc., (Seoul, South Korea) and maintained in a laminar air-flow cabinet under specific pathogen-free conditions. A549 cells were transduced with control shRNA virus (shCTR) or BRD7 shRNA virus (shBRD7) and a 0.1 ml suspension of each containing 1 × 10^6^ cells was subcutaneously injected into the right flank of each mouse. When the tumor size reached 150 mm^3^ for both the shCTR and shBRD7 groups, the mice treated with each shRNA were randomly divided into two groups (10 mice/group). The experimental group of mice received an intratumoral injection of vehicle or interferon-γ (IFN-γ, 1 × 10^5^ international units [IU]) solution three times a week. Two perpendicular diameters of the tumors were measured three times per week with a caliper square by the same investigator, and the tumor volume was calculated using the following equation: Tumor volume (V) mm^3^ = (smaller diameter)^2^ x (larger diameter) x (π/6). The experiment was terminated 5 weeks after tumor cell implantation because the tumors in the control mice began to show signs of possible necrosis. The use of these animals and the experimental procedures were approved by the Institutional Animal Care and Use Committee of the Korea Institute of Radiological and Medical Sciences.

### Statistical analysis

All of the data are presented as the mean ± SD. Student's two-tailed *t*-test was employed for all of the analyses, and a *p* value less than 0.05 was considered statistically significant.

## Abbreviations

HMVEC: human pulmonary microvascular endothelial cell
XAF1: X-linked inhibitor of apoptosis-associating factor 1
BRD7: bromodomain 7
IFN-γ: Interferon-gamma
IRF-1: Interferon regulatory factor-1
PD: population doublings
SA-β-gal: senescence-associated β-galactosidase.
